# *ALDH7A1* rs12514417 polymorphism may increase ischemic stroke risk in alcohol-exposed individuals

**DOI:** 10.1186/s12986-022-00702-3

**Published:** 2022-10-18

**Authors:** Chun-Hsiang Lin, Oswald Ndi Nfor, Chien-Chang Ho, Shu-Yi Hsu, Disline Manli Tantoh, Yi-Chia Liaw, Daria Mochly-Rosen, Che-Hong Chen, Yung-Po Liaw

**Affiliations:** 1 Department of Public Health, Institute of Public Health, Chung Shan Medical University, 40201 Taichung City, Taiwan; 2 Department of Neurology, Yuanlin Christian Hospital, Changhua County 510, Yuanlin, Taiwan; 3 Department of Chemical and Systems Biology, Stanford University School of Medicine, 94305 Stanford, CA USA; 4 Department of Physical Education, Fu Jen Catholic University, 24205 New Taipei City, Taiwan; 5 Research and Development Center for Physical Education, Health and Information Technology, Fu Jen Catholic University, 24205 New Taipei City, Taiwan; 6 Department of Medical Education, Taipei Veterans General Hospital, 11217 Taipei City, Taiwan; 7 Department of Medical Imaging, Chung Shan Medical University Hospital, 40201 Taichung City, Taiwan; 8 Institute of Medicine, Chug Shan Medical University, 40201 Taichung City, Taiwan

**Keywords:** Cardiovascular disease, Stroke, Epidemiology, *ALDH7A1*, Polymorphism

## Abstract

**Background:**

Epidemiological studies have identified common risk factors for cerebral stroke worldwide. Some of these factors include hypertension, diabetes, smoking, excessive drinking, and dyslipidemia. It is important to note, however, that genetic factors can also contribute to the occurrence of stroke. Here, we evaluated the association of ischemic stroke with rs12514417 polymorphism of the alcohol metabolizing gene, aldehyde dehydrogenase 7A1 (*ALDH7A1*) and alcohol consumption.

**Methods:**

Taiwan Biobank (TWB) data collected between 2008 and 2015 were available for 17,985 subjects. The odd ratios for stroke were obtained using logistic regression models.

**Results:**

Among eligible subjects (n = 17,829), 897 had ischemic stroke and 70 had hemorrhagic stroke. Subjects with ischemic stroke were older (mean ± SE, 58.45 ± 8.19 years vs. 48.33 ± 10.89 years, p < 0.0001) and had a higher body mass index (BMI) than the stroke-free individuals. The risk of ischemic stroke was significantly higher among subjects with the *ALDH7A1* rs12514417 TG + GG genotype who also consumed alcohol at least 150 ml/week (odds ratio (OR), 1.79; 95% confidence interval (CI), 1.18–2.72). We found that rs12514417 genotype and alcohol consumption (at least 150 ml/week) showed a significant interaction (p for interaction = 0.0266). Stratification based on alcohol exposure and *ALDH7A1* rs12514417 genotypes indicated that ischemic stroke risk was significantly higher among alcohol drinkers with the TG + GG genotype than in those with the TT genotype (OR, 1.64, 95% CI: 1.15–2.33).

**Conclusion:**

Our study suggests that the combination of *ALDH7A1* rs12514417 TG + GG genotype and alcohol exposure of at least 150 ml/week may increase the risk of ischemic stroke in Taiwanese adults.

## Introduction

About 12 million people are affected by stroke worldwide[1]. In spite of being the second leading cause of death after coronary artery disease (CAD), it is associated with more disabilities than CAD [2]. Over 87% of all strokes are ischemic, with the remaining strokes being hemorrhagic, including cerebral and subarachnoid hemorrhages. There are a number of modifiable risk factors for stroke, including hypertension, smoking, diabetes, hyperlipidemia, high salt intake, binge alcohol drinking, high-fat diets, and inactivity [3]. However, the relationships between alcohol consumption and stroke vary between epidemiological studies. According to a meta-analysis on alcohol drinking and stroke types by Larsson et al., [4] light (less than 1 drink/day) and moderate (1–2 drinks/day) alcohol consumption were inversely associated with ischemic stroke, whereas heavy drinking (> 4 drinks/day) was associated with increased risk of all stroke types, with hemorrhagic stroke showing the strongest association.

Alcohol metabolism is genetically controlled and can significantly affect drinking behavior and alcohol-related organ damage [5]. Alcohol is first converted to acetaldehyde by alcohol dehydrogenase 1B (*ADH1B*). Acetaldehyde is further oxidized by aldehyde dehydrogenase (*ALDH*) isoenzymes to nontoxic acetate, using NAD + as a cofactor[6, 7]. *ALDH2* is considered one of the most important enzymes for ethanol metabolism in vivo, and an inactivating mutation in *ALDH2*, the common East Asian ALDH2*2 (the rs671 E504K missense variant), is associated with a wide range of health complications, such as osteoporosis, cancer (about 20% increased cancer risk in inactive, ALDH2*2, homozygous individuals) and Alzheimer’s disease [8–10]. In addition, several studies suggest that *ALDH2* protects against oxidative stress and could influence the onset of hypertension[11, 12]. ALDH2*2 has a lower NAD + coenzyme binding affinity, which reduces the clearance capacity of acetaldehyde, thereby increasing the risk of ischemic stroke[6].

We have previously reported that the *ADH1B* rs1229984 variant is associated with hemorrhagic stroke among alcohol drinkers, while the ALDH2*2 variant showed no association with either ischemic or hemorrhagic stroke among alcohol drinkers[13].

Chronic alcoholism frequently results in specific micronutrient deficiencies and can interfere with one-carbon metabolism, for which vitamin B12, B6, and folate all act as coenzymes[14]. In chronic alcoholics, serum pyridoxal 5’-phosphate (an active form of vitamin B6) and red blood cell folate concentrations were found to be significantly lower than in control subjects[14]. Following a 2-week intervention study with vodka or red wine, serum vitamin B12 and folate levels significantly decreased [15]. Folate, vitamin B6, and vitamin B12 deficiency are known to elevate homocysteine levels in blood [14, 16], which, in turn, may increase the risk of ischemic stroke.[17]. In addition, elevated levels of homocysteine in acute stroke were related to higher mortality.[18].

As a member of the *ALDH* gene family, Antiquitin *(ALDH7)* is not only involved in ethanol metabolism but also has α-aminoadipic semialdehyde (α-AASA) dehydrogenase activity [19]. Mutations of the antiquitin gene result in reduced pyridoxal-5-phosphate (PLP) activity and cause pyridoxine-dependent seizure, a recessive disorder that responds dramatically to the intravenous injection of pyridoxine (vitamin B6). Considering the importance of *ALDH7A1* in both vitamin B6 and ethanol metabolism, we examined the potential correlations between the most common polymorphic locus in *ALDH7A1* among East Asians (rs12514417; Lys439Gln missense variant allele frequency 12.9% in Asian [20]), stroke, and alcohol consumption.

## Methods

### Data source

We included data from two data resources: (1) The National Health Insurance Research Database (NHIRD), with medical data available from 1998 to 2015 and (2) Taiwan Biobank, a national health resource that is open to researchers and contains basic demographic and genotype data on ethnic Taiwanese residents (aged 30 to 70 years) from 2008 to 2015. The biobank aims to facilitate the development of better prevention and treatment strategies for chronic diseases such as cerebrovascular disease, cancer, liver cirrhosis, or other conditions listed among the ten leading causes of death in Taiwan. We linked the two databases at the Health and Welfare Data Science Center (HWDC) and obtained information about stroke occurrences through personal identification numbers. The Institutional Review Board of Chung Shan Medical University Hospital (CS1-20009) approved this study.

### Patient identification

The current analyses included 17,985 TWB subjects. The information included sex, age, body mass index (BMI), lifestyle exposures (regular exercise, smoking, and alcohol consumption), and the rs12514417 polymorphism. The outcome variable was an ischemic stroke. We excluded subjects with incomplete questionnaires and genotyping data (n = 103), those with both ischemic and hemorrhagic stroke (n = 41) as well as those who started to drink after developing stroke (n = 12). After the exclusions, the final enrollment included 897 patients with ischemic stroke, 70 hemorrhagic stroke patients, and 16,862 stroke-free controls.

We defined alcohol drinkers as persons who reported drinking more than 150 ml of alcohol per week during the past six months and were still drinking during assessment visits. Exercise included any amount of physical activity at least 3 times a week and lasting for at least 30 min each time. Other lifestyle exposures have already been discussed in our previous publication [21].

### Genetic variant selection

A literature search using Pub Med, ScienceDirect, Google Scholar, SNPedia, and the GWAS Catalog was carried out to identify common *ALDH7A1* gene variants. The most common polymorphism, rs12514417, was chosen after searching these databases [20]. SNP genotyping was carried out using the custom TWB chips and run on the Axiom™ Genome-Wide Array Plate System (Affymetrix, Santa Clara, CA, USA). The rs12514417 polymorphism passed the quality control test: the call rate was > 95%, the p-value for the Hardy-Weinberg equilibrium test was > 1.0 × 10^− 3^, and the minor allele frequency was > 0.05.

### Definition of outcomes

Patients were defined as having an ischemic or hemorrhagic stroke if they had either two outpatient visits or one-time hospitalization from 1998 to 2015 with reported International Classification of Diseases, Ninth Revision, Clinical Modification (ICD-9 CM) 433–437 and 430–432, respectively. Potential covariates included hypertension (ICD-9-CM: 401–405), diabetes mellitus (ICD-9-CM: 250), and hyperlipidemia (ICD-9-CM: 272). Since metabolic syndrome is more prevalent among those with stroke, we added the metabolic syndrome in the model to analyze. Participants with any three of the following risk factors were considered to have metabolic syndrome: (1) central obesity (waist circumference (WC)  90 cm for men, ≥80 cm for women); (2) high blood pressure (SBP≥ 130 mmHg and/or DBP ≥85mmHg); (3) low HDL-C (HDL-C < 40 mg/dL for men, < 50 mg/dL for women); (4) increased fasting plasma glucose ( ≥ 100 mg/dL); and (5) elevated TG level ( ≥ 150 mg/dL).

### Statistical analysis

We used the PLINK 1.09 beta and SAS 9.4 software (SAS Institute, Cary, NC) for data management and statistical analyses. We performed descriptive statistics using chi-square and t-tests. We ran logistic regression models to determine the ORs and 95% CI for developing ischemic stroke/hemorrhagic stroke among the rs12514417 individuals based on alcohol exposure. We also used logistic regression analysis to determine the interaction between alcohol exposure and* ALDH7A1 *rs12514417 on ischemic stroke/hemorrhagic, followed by stratified analyses based on these variables. The covariates included the sex, age, education, smoking, physical activity, BMI, diabetes, hypertension and hyperlipidemia. A two-sided P value less than 0.05 was considered statistically significant.

## Results

The basic characteristics of the study subjects are shown in Table 1. The study comprised 897 individuals with ischemic stroke, 70 with hemorrhagic stroke, and 16,862 with neither, whom we refer to as control subjects. Subjects with ischemic stroke were older (mean ± SE, 58.45 ± 8.19 years vs. 48.33 ± 10.89 years, p < 0.0001) and had a higher body mass index (BMI) than control individuals. The distributions of risk factors for cerebrovascular disease such as hypertension, hyperlipidemia, and diabetes were significantly different between cases and controls (p < 0.0001, Table 1). We found that *ALDH7A1* rs12514417 was not associated with hemorrhagic stroke based on alcohol intake (Table 2). However, importantly, the risk for ischemic stroke was higher among carriers of the *ALDH7A1*, rs12514417 TG + GG who drank alcohol (OR, 1.70; 95% CI, 1.16–2.50). In addition, significant associations with ischemic stroke were found for hypertension and hyperlipidemia among patients with both rs12514417 TT and TG + GG genotypes. Among subjects with the TT genotype, the OR (95% CI) was 1.54 (1.27–1.87), 2.53 (2.07–3.09), and 1.62 (1.32–1.98) for those with diabetes, hypertension, and hyperlipidemia, respectively. There was an interaction between alcohol consumption and rs12514417 (p = 0.0266).

**Table 1:** Descriptive data of the subjects

	Control n = 16,862		Ischemic n = 897		Hemorrhagic n = 70		p-value^a^	p-value^b^
	**N (%)**		**N (%)**		**N (%)**			
*ALDH7A1* rs12514417							0.316	0.212
TT	11,959	(70.92)	615	(68.56)	54	(77.14)		
TG	4496	(26.66)	259	(28.87)	13	(18.57)		
GG	407	(2.41)	23	(2.56)	3	(4.29)		
Sex							0.099	0.211
Female	8729	(51.77)	439	(48.94)	31	(44.29)		
Male	8133	(48.23)	458	(51.06)	39	(55.71)		
Smoking							0.517	0.103
No	12,737	(75.54)	669	(74.58)	47	(67.14)		
Yes	4125	(24.46)	228	(25.42)	23	(32.86)		
Alcohol intake							0.085	0.022
No	15,135	(89.76)	789	(87.96)	57	(81.43)		
Yes	1727	(10.24)	108	(12.04)	13	(18.57)		
Diabetes							< 0.001	0.008
No	14,783	(87.67)	573	(63.88)	54	(77.14)		
Yes	2079	(12.33)	324	(36.12)	16	(22.86)		
Hypertension							< 0.001	0.008
No	13,269	(78.69)	353	(39.35)	46	(65.71)		
Yes	3593	(21.31)	544	(60.65)	24	(34.29)		
Hyperlipidemia							< 0.001	0.007
No	12,087	(71.68)	326	(36.34)	40	(57.14)		
Yes	4775	(28.32)	571	(63.66)	30	(42.86)		
Age (mean ± SD)	48.32	± 10.89	58.45	± 8.19	50.66	± 10.64	< 0.001	0.073
BMI (mean ± SD)	24.23	± 3.63	24.83	± 3.65	24.51	± 4.16	< 0.001	0.531

**Table 2:** Association of ischemic and hemorrhagic stroke with alcohol intake in rs12514417 individuals

	Ischemic stroke				Hemorrhagic stroke			
	**rs12514417(TT)**		**rs12514417(TG + GG)**		**rs12514417(TT)**		**rs12514417(TG + GG)**	
	**OR**	**(95% CI)**	**OR**	**(95% CI)**	**OR**	**(95% CI)**	**OR**	**(95% CI)**
Alcohol intake (ref: No)								
Yes	0.85	(0.64–1.15)	1.7	(1.16–2.50)	1.9	(0.86–4.05)	1.1	(0.29–4.36)
Sex (ref: Female)								
Male	0.99	(0.81–1.22)	1.1	(0.81–1.50)	1	(0.53–1.98)	2.2	(0.61–7.80)
Age	1.07	(1.06–1.08)	1.1	(1.06–1.10)	1	(0.98–1.04)	1	(0.93–1.04)
Education (ref: Elementary School)								
Junior & Senior High School	1.01	(0.77–1.32)	1.3	(0.84–1.99)	0.7	(0.24–1.85)	1.7	(0.20-14.18)
University & above	0.8	(0.60–1.07)	1.2	(0.73–1.81)	0.6	(0.23–1.83)	0.6	(0.06–5.40)
Smoking (ref: No)								
Yes	1.04	(0.83–1.32)	0.7	(0.52–1.03)	1.1	(0.52–2.20)	1.4	(0.42–4.46)
Physical activity (ref: No)								
Yes	1.08	(0.90–1.29)	1	(0.77–1.30)	0.7	(0.39–1.26)	1.5	(0.51–4.19)
BMI	1.01	(0.98–1.04)	1	(0.94–1.01)	1	(0.88–1.04)	1	(0.90–1.18)
Diabetes (ref: No)								
Yes	1.54	(1.27–1.87)	1.3	(0.96–1.75)	0.8	(0.33–1.72)	9	(2.54–32.09)
Hypertension (ref: No)								
Yes	2.53	(2.07–3.09)	1.9	(1.45–2.59)	1.5	(0.76–2.98)	1	(0.27–3.46)
Hyperlipidemia (ref: No)								
Yes	1.62	(1.32–1.98)	1.7	(1.27–2.27)	1.7	(0.90–3.27)	0.7	(0.19–2.29)

Alcohol intake*rs12514417 in Ischemic stroke, p = 0.0266.; Alcohol intake*rs12514417 in Hemorrhagic stroke, p = 0.9101

Furthermore, we wanted to see if genes would influence the stroke risk among drinkers. After stratification by alcohol consumption (Table 3), the odds of having ischemic stroke remained significantly higher among *ALDH7A1* rs12514417 TG + GG subjects who consumed alcohol (OR = 1.80, 95% CI: 1.19–2.73). No significant association was found between the rs12514417 genotype and hemorrhagic stroke risk regardless of whether the subjects consumed alcohol or not. Using the TT genotype and no alcohol intake as a reference group (Table 4), the risk for ischemic stroke was significantly higher in people with TG + GG genotype who consumed alcohol (OR = 1.67; 95% CI: 1.18–2.38).

**Table 3:** Association of ischemic and hemorrhagic stroke with rs12514417 based on alcohol intake

	Ischemic stroke				Hemorrhagic stroke			
	**No alcohol intake**		**Alcohol intake**		**No alcohol intake**		**Alcohol intake**	
	**OR**	**(95% CI)**	**OR**	**(95% CI)**	**OR**	**(95% CI)**	**OR**	**(95% CI)**
Rs12514417(ref: TT)								
TG + GG	1.08	(0.92–1.27)	1.79	(1.18–2.72)	0.72	(0.39–1.33)	0.83	(0.23–3.08)
Sex (ref: Female)								
Male	1	(0.84–1.19)	1.33	(0.62–2.85)	1.35	(0.73–2.48)	0.43	(0.11–1.69)
Age	1.07	(1.06–1.08)	1.04	(1.02–1.07)	1.01	(0.98–1.05)	0.96	(0.90–1.02)
Education (ref: Elementary School)								
Junior & Senior High School	1.11	(0.87–1.41)	0.84	(0.40–1.76)	1.12	(0.38–3.33)	0.25	(0.04–1.41)
University & above	0.93	(0.72–1.20)	0.67	(0.31–1.45)	0.9	(0.29–2.77)	0.13	(0.02–0.96)
Smoking (ref: No)								
Yes	0.97	(0.79–1.21)	0.71	(0.46–1.11)	1.2	(0.61–2.34)	0.94	(0.27–3.27)
Physical activity (ref: No)								
Yes	1.08	(0.92–1.27)	0.83	(0.54–1.29)	0.69	(0.38–1.22)	1.9	(0.59–6.17)
BMI	1	(0.98–1.03)	0.97	(0.91–1.03)	0.99	(0.92–1.07)	0.93	(0.78–1.10)
Diabetes (ref: No)								
Yes	1.48	(1.25–1.76)	1.48	(0.93–2.35)	1.28	(0.62–2.64)	2.88	(0.69–12.10)
Hypertension (ref: No)								
Yes	2.25	(1.89–2.68)	3.03	(1.85–4.96)	1.32	(0.68–2.56)	1.61	(0.39–6.66)
Hyperlipidemia (ref: No)								
Yes	1.63	(1.37–1.94)	1.63	(1.02–2.61)	1.55	(0.82–2.92)	0.87	(0.21–3.68)

Abbreviations: OR = odds ratio, CI = 95% confidence interval, BMI = body mass index, TT, TG, and GG = rs12514417 genotypes

**Table 4:** Association of ischemic and hemorrhagic stroke with alcohol intake and rs12514417 genotypes

	Ischemic stroke		Hemorrhagic stroke	
	**OR**	**(95% CI)**	**OR**	**(95% CI)**
TT, alcohol intake	0.91	(0.68–1.22)	1.59	(0.75–3.38)
TG + GG, no alcohol intake	1.08	(0.92–1.27)	0.72	(0.39–1.34)
TG + GG, alcohol intake	1.64	(1.15–2.33)	1.24	(0.37–4.17)
Sex (ref: Female)				
Male	1.03	(0.87–1.22)	1.19	(0.67–2.11)
Age	1.07	(1.06–1.08)	1	(0.98–1.03)
Education (ref: Elementary School)				
Junior & Senior High School	1.08	(0.86–1.36)	0.85	(0.34–2.10)
University & above	0.89	(0.70–1.14)	0.64	(0.25–1.66)
Smoking (ref: N0)				
Yes	0.93	(0.77–1.13)	1.14	(0.62–2.11)
Physical activity (ref: No)				
Yes	1.05	(0.91–1.22)	0.83	(0.50–1.38)
BMI	1	(0.98–1.02)	0.98	(0.91–1.05)
Diabetes (ref: No)				
Yes	1.47	(1.25–1.74)	1.47	(0.77–2.79)
Hypertension (ref: No)				
Yes	2.33	(1.98–2.74)	1.37	(0.75–2.49)
Hyperlipidemia (ref: No)				
Yes	1.64	(1.39–1.93)	1.42	(0.80–2.54)

Abbreviations: OR = odds ratio, CI = 95% confidence interval, BMI = body mass index, TT, TG, and GG = rs12514417 genotypes

## Discussion

The pathogenesis of cerebral stroke is complex; various risk factors of for cerebral stroke identified by epidemiological studies mainly included aging, hypertension, hyperlipidemia, diabetes, smoking, excessive drinking, among others[1].

Stroke is also likely to be caused by genetic factors; a synergistic effect of genetic and environmental factors can lead to stroke in some individuals. Combining two data sources, we demonstrated the relationship between stroke and the rs12514417 polymorphism and its impact on alcohol consumption. Our main finding suggests that the *ALDH7A1* rs12514417 TG + GG genotype may be associated with a 1.6-fold increased risk of ischemic stroke in subjects who drink more than 150 ml of alcohol per week[6, 7, 22–24]. This is the first study to identify the relationship between *ALDH7A1* mutation and ischemic stroke, suggesting a change in lifestyle, specifically a reduction in alcohol consumption could reduce disease risk in carriers of this variant.

Studies have found a link between stroke and *ALDH2*, another aldehyde dehydrogenase [6, 7, 22–24]. *ALDH2* is the most studied aldehyde dehydrogenase[25], which are associated with decreased enzymatic activity, liver disease, cirrhosis, or pancreatitis in alcoholics[25]. Studies have suggested that the ALDH2*2 allele is an important risk factor for ischemic stroke in Taiwanese [23] and Korean [26] men, as well as in Chinese women (OR = 2.207, 95% CI 1.416–3.439)[22], since it increases dyslipidemia, hypertension, and diabetes, which may contribute to cerebral arteriosclerosis [27–29].

Besides the inflammatory mechanism, oxidative stress plays a critical role in the development of ischemic stroke. As oxidative stress develops, most aldehydes are produced by lipid peroxidation. Reactive aldehydes can further cause structural damage to biological macromolecules, such as DNA, lipids, and proteins caused by reactive oxygen species, including 4-Hydroxynonenal (4-HNE), malondialdehyde (MDA) and others [30]. The *ALDH2* protein can detoxify these reactive aldehydes and is critical to the health of cells and therefore serves as an important shield against damage occurring under oxidative stress[30]. A previous studies have shown that expression of 4-HNE-protein was elevated in the ischemic cerebral cortex when measured within two hours of stroke induction in genetically stroke-prone mice [31]. Moreover, Lee et al. [32] demonstrated that plasma 4-HNE levels were elevated in patients with stroke and genetic stroke-prone mice (stroke-prone spontaneously hypertensive rats), as well as in experimental stroke rats with middle cerebral artery occlusion (MCAO). Intravenous administration of 4-HNE before stroke not only enlarged the cerebral ischemia-induced infarct area but also increased oxidative stress in mice. Transgenic mice overexpressing *ALDH2* after 12 weeks of alcohol consumption displayed significantly reduced levels of brain damage and neuronal cell death [33]. To sum up the above, mutation of the *ALDH2* gene (ALDH2*2), which results in a reduced enzyme activity, may increase oxidative stress and cause cerebral damage[33].

Unlike ALDH2, the function of* ALDH7A1* is not well understood and no literature has described its association with stroke so far. *ALDH7A1* is also considered to play a role in detoxifying aldehydes generated by alcohol metabolism and lipid peroxidation. Although most ALDH isozymes are expressed exclusively in the liver, extra-hepatic tissues may also express a relatively large amount of a specific *ALDH* isozyme, depending on their specific functions[34]. According to tissue distribution studies in mice, *ALDH7A1* was most highly expressed in the brain, liver, and kidney [35]. The *ALDH* organ-specific distribution may indicate a greater likelihood of neurotoxicity caused by reduced activity of *ALDH7A1* compared with *ALDH2*. Perhaps a more important function of *ALDH7A1* in relation to stroke risk is its role in lysine catabolism through the oxidation of AASA. Lysine catabolism is essential for maintaining cellular nitrogen stores and for producing ketone bodies(KBs) [36]. KBs are an important source of energy for the brain in a nutrient deprivation state. Growing evidence indicates that ketone bodies have beneficial effects on stroke treatment, but the mechanisms are not clear[37]. After transient middle cerebral artery (MCA) occlusion in mice, ketones improved mitochondrial function and reduced oxidative stress by promoting NAD+-dependent Sirtuin 3 (SIRT3) and its downstream substrates in the penumbra, thereby reducing the infarct volume and improving neurological function after ischemic stroke [38].

**Fig. 1:**
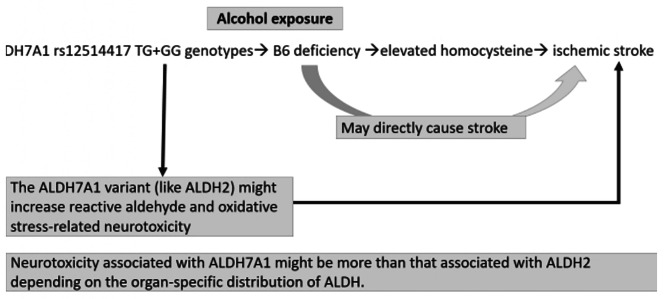
A possible mechanism through which the rs12514417 TG + GG genotype and alcohol consumption may lead to stroke

Homozygous mutations in *ALDH7A1* decrease antiquitin acitivity, which can result in pyridoxine-dependent and folic acid-responsive seizure[39]. Deficiency of this activity leads to the accumulation of AASA and piperideine-6-carboxylate (P6C), the coenzyme form of vitamin B6 which activates PLP, leading to lifelong pyridoxine (Vit B6) demands[40]. Individuals with chronic alcoholism may further exhibit lower plasma levels of PLP, made even more severe by vitamin B6 deficiency[41]. Homocysteine metabolism-related vitamins (HMRV), including Vitamin B6, folic acid, and vitamin B12 are all cofactors in homocysteine metabolism. Vitamin B6 deficiency may elevate plasma homocysteine[42, 43]. There is growing evidence to suggest that hyperhomocysteinaemia is a risk factor for stroke, especially among hypertensive individuals[17]. Studies have suggested that hyperhomocysteinemia is closely correlated with ischemic stroke, as it induces atherosclerosis through the pathophysiologic mechanisms of intracranial small-vessel and extracranial large-artery diseases[44–46]. The severity of carotid atherosclerosis was inversely associated with plasma concentrations of folate and PLP after adjusting for age, sex, and other risk factors[44]. HMRV supplementation may lower the incidence of stroke-induced hyperhomocysteinemia resulting from HMRV deficiency. In a randomized controlled trial (HOPE-2 study)[47], HMRV supplementation combined with folic acid, vitamins B6 and B12 reduced the risk of stroke by 25%. Despite these, more studies are needed to ascertain whether homocysteine, folic acid, and vitamins B6 and B12 metabolism differ among subjects carrying the *ALDH7A1* variant. A summary of possible mechanisms through which the rs12514417 TG + GG genotype and alcohol consumption may result in stroke is shown in Fig. 1.

The main limitation of our study is the uncertainty about alcohol exposure. The amount of alcohol consumed was measured in millimeters (ml), rather than milligrams (mg). Data on alcohol consumption were self-reported and are subject to recall bias. The second limitation is the lack of homocysteine data for those subjects. However, vitamin B6 deficiency could be an independent risk factor for ischemic stroke in the presence or absence of hyperhomocysteinemia. According to Kelly et al., low PLP was associated with chronic inflammation and elevated C-reactive protein (CRP) in addition to the acute phase response. There was no association between CRP and homocysteine, despite a significant correlation between homocysteine and PLP[48]. Clinical evidence demonstrated that low levels of vitamin B6, but not homocysteine were strongly associated with ischemic stroke and transient ischemic attack[49, 50].

The main finding of our study suggests that the *ALDH7A1* rs12514417 TG + GG genotype is associated with a 1.6-fold increased risk of ischemic stroke in subjects who drink more than 150 ml of alcohol per week. The underlying mechanism is that *ALDH7A1* mutations can cause oxidative stress, inflammation, and vitamin B6 deficiency and therefore result in ischemic stroke. It remains to be determined if the common rs12514417; Lys439Gln missense mutation would cause any change in the enzymatic function of *ALDH7A1* in vitro and in vivo. We suggest that a large study on gene-environment interactions in diverse populations be conducted and also functional assessment using animal models or human cell lines to examine the underlying mechanisms behind this potential association.

## Data Availability

The data that support the findings of this study are available from the Taiwan Biobank data source but restrictions apply to the availability of these data, which were used under license for the current study, and so are not publicly available. Data are however available from the authors upon reasonable request and with permission of Taiwan Biobank.

## Abbreviations

ALDH7A1: aldehyde dehydrogenase 7A1
ALDH: Aldehyde dehydrogenase
ADH1B: Alcohol dehydrogenase 1B
TWB: Taiwan Biobank
NHIRD: National Health Insurance Research Database
MOST: Ministry of Science and Technology
CAD: Coronary artery disease
BMI: Body mass index
HWDC: Health and Welfare Data Science Center
SE: Standard error
OR: Odds ratio
CI: Confidence interval
